# Identification of Epileptogenic and Non-epileptogenic High-Frequency Oscillations Using a Multi-Feature Convolutional Neural Network Model

**DOI:** 10.3389/fneur.2021.640526

**Published:** 2021-10-15

**Authors:** Guoping Ren, Yueqian Sun, Dan Wang, Jiechuan Ren, Jindong Dai, Shanshan Mei, Yunlin Li, Xiaofei Wang, Xiaofeng Yang, Jiaqing Yan, Qun Wang

**Affiliations:** ^1^Department of Neurology, Beijing Tiantan Hospital, Capital Medical University, Beijing, China; ^2^China National Clinical Research Center for Neurological Diseases, Beijing, China; ^3^Collaborative Innovation Center for Brain Disorders, Beijing Institute of Brain Disorders, Capital Medical University, Beijing, China; ^4^Department of Neurology, Xingtai People's Hospital, Hebei, China; ^5^Department of Functional Neurosurgery, Beijing Haidian Hospital, Beijing, China; ^6^Department of Neurology, Xuanwu Hospital, Capital Medical University, Beijing, China; ^7^Department of Neurosurgery, Capital Institute of Pediatrics, Children's Hospital, Beijing, China; ^8^Department of Neurology, National Center for Children's Health, Beijing Children's Hospital, Capital Medical University, Beijing, China; ^9^Guangzhou Laboratory, Guangzhou, China; ^10^College of Electrical and Control Engineering, North China University of Technology, Beijing, China

**Keywords:** high frequency oscillations, epileptogenic zone, convolutional neural network, adjusted smoothed pseudo Wigner–Ville distribution, refractory focal epilepsy

## Abstract

Accurately identifying epileptogenic zone (EZ) using high-frequency oscillations (HFOs) is a challenge that must be mastered to transfer HFOs into clinical use. We analyzed the ability of a convolutional neural network (CNN) model to distinguish EZ and non-EZ HFOs. Nineteen medically intractable epilepsy patients with good surgical outcomes 2 years after surgery were studied. Five-minute interictal intracranial electroencephalogram epochs of slow-wave sleep were selected randomly. Then 5 s segments of ripples (80–200 Hz) and fast ripples (FRs, 200–500 Hz) were detected automatically. The EZs and non-EZs were identified using the surgery resection range. We innovatively converted all epochs into four types of images using two scales: original waveforms, filtered waveforms, wavelet spectrum images, and smoothed pseudo Wigner–Ville distribution (SPWVD) spectrum images. Two scales were fixed and fitted scales. We then used a CNN model to classify the HFOs into EZ and non-EZ categories. As a result, 7,000 epochs of ripples and 2,000 epochs of FRs were randomly selected from the EZ and non-EZ data for analysis. Our CNN model can distinguish EZ and non-EZ HFOs successfully. Except for original ripple waveforms, the results from CNN models that are trained using fixed-scale images are significantly better than those from models trained using fitted-scale images (*p* < 0.05). Of the four fixed-scale transformations, the CNN based on the adjusted SPWVD (ASPWVD) produced the best accuracies (80.89 ± 1.43% and 77.85 ± 1.61% for ripples and FRs, respectively, *p* < 0.05). The CNN using ASPWVD transformation images is an effective deep learning method that can be used to classify EZ and non-EZ HFOs.

## Introduction

Pathological high frequency oscillations (HFOs) have been proposed as a promising biomarker for the epileptogenic zone (EZ) (1). They are defined as four continuous oscillations within the 80–500 Hz range whose amplitudes are significantly higher than the baseline. They are commonly divided into ripples (80–200 Hz) and fast ripples (FRs, 200–500 Hz) (2). Resection of areas with high HFO occurrence ratios is associated with good surgical outcomes (3). In addition, there is an ongoing multi-center study that compares the clinical values of interictal HFOs with the epileptogenicity index and interictal/preictal functional connectivity alterations for surgical decision making in patients with focal cortical dysplasia (4). HFOs (especially ripples) can be recorded both inside and outside the seizure onset zone (SOZ) or EZ (5, 6). HFOs located in the hippocampus, sensorimotor cortex, or occipital lobe may be related to physiological activities (7, 8) such as memory consolidation (9) or visual perceptual learning (10). The existence of physiological HFOs influence the accuracy to identify EZs using HFOs in some brain areas. In addition, FRs outside the EZ disappear concurrently after resection of FRs inside the EZ in patients with good surgical outcomes. This indicates the presence of an epileptogenic network (11). Our previous retrospective study also found that not all of the brain tissues that produce HFOs need to be removed completely for good surgical outcomes (12). Therefore, subsequent studies focus on further development of novel methods to classify EZ and non-EZ HFOs to delineate the EZ range accurately.

Deep learning is a type of algorithm that automatically establishes rules for classifying data and uses these rules to predict unknown data (13). These algorithms involve probability theory, statistics, approximation theory, convex analysis, algorithmic complexity theory, etc. They are developed using artificial neural networks, which form abstract high-level features by combining low-level features to discover distributed feature representations in data. Deep learning has been applied to image recognition, speech recognition, brain circuits reconstructing, and many other areas of scientific research (13). It uses various structures such as deep confidence networks (DBNs) and convolutional neural networks (CNNs), that can express a variety of data with different relations between dimensions. CNNs have been applied to electrophysiological data analysis. They perform well in decoding of task-related electroencephalogram (EEG) data and classification of EEG signals in various brain–computer interface tasks (14–16). Furthermore, CNNs have been implemented for automatic HFOs detection (17–19). However, the use of CNNs to differentiating EZ and non-EZ HFOs has not been studied. Therefore, this retrospective study used a multi-feature CNN model to perform deep learning of EZ and non-EZ HFO characteristics and established a corresponding mathematical classification model to assist in surgical decision making.

## Materials and Methods

### Patient Population

The patient inclusion criteria consisted of the following: (1) intractable epilepsy patients that were addressed via EZ removal surgery at the Epilepsy Centre of Beijing Haidian Hospital between January 2013 and December 2015; (2) implantation of subdural grids or depth electrodes followed by intracranial EEG monitoring with video at a sampling rate of 2,000 Hz for at least one entire night; and (3) at least 2 years of patient follow-up after surgery to confirm an Engel I surgical outcome. Patients with serious EEG artifacts or a lack of surgical data were excluded.

Patients over 18 years of age and the legal guardian or next of kin for those under 18 gave informed consent in agreement with the requirements dictated by the Research Ethics Board of Beijing Haidian Hospital. Patients were still under antiepileptic drug therapy at the time of the recording. However, the drug doses might have been tapered in some patients to induce seizures so that the SOZ could be identified.

### Electrode Placement and Intracranial Electroencephalogram Recording

Several types of electrodes were implanted in putative epileptogenic areas based on previous non-invasive pre-surgical evaluations. A combination of cortical strips, grid electrodes (contact diameter 4 mm with 2.5 mm of exposure and 10 mm of spacing between contact centers; Beijing Huakehengsheng Healthcare Co., Ltd., Beijing, China), and mesiotemporal depth electrodes (1.2 mm diameter, eight 2 mm-long contacts, 10 mm between contacts; Beijing Huakehengsheng Healthcare Co., Ltd., Beijing, China) were implanted. Preimplantation magnetic resonance imaging (MRI) and post-implantation computer tomography (CT) scans were used to locate each contact anatomically along the electrode trajectory.

Data were recorded starting on the day after electrode implantation. Data for HFO analysis were acquired at 2,000 Hz using a 32- or 256-channel Nicolet recording system (Natus Medical Incorporated, San Carlos, CA, United States). The recordings were performed in a monitoring unit that was under video surveillance.

### Delineation of Epileptogenic Zone and Non-epileptogenic Zone

The EZ was identified by neurologists and neurosurgeons based on long-term intracranial EEG monitoring. The resected channels were confirmed by comparing the fusion of pre-surgical MRI and CT with post-surgical MRI. EZ was defined as the area of the cortex that generates seizures, and which should be removed to make the patient seizure-free. In this study, all of the included patients had Engel I surgical outcomes at least 2 years after surgery. Therefore, we considered the tissues that were resected to be the EZ and the non-resected areas to be non-EZ tissues. All electrodes in EZ and non-EZ were included for analysis.

### Data Selection and High-Frequency Oscillations Detection

We chose a slow-wave sleep segment as this period includes less muscle activity and more HFOs than other periods. We used the same method as several previous studies (12, 20). We then selected a random 5 min segment during the slow wave sleep period of each patient. All data were selected from interictal periods that occurred at least 2 h from a seizure. Data with artifacts or noise such as sharp transients with absolute amplitudes >6 standard deviations (SD) from the baseline mean amplitude or irregular signals, were not selected. The data were transformed into a bipolar montage for further analysis. Our preliminary algorithm automatically detected HFOs based on maximum peak points (12, 21). “False HFOs” that were probably caused by filtering of spikes or sharp transients were automatically deleted. Because ripples and FRs have different generation mechanisms and electrophysiological characteristics, the algorithm was designed to analyze the two types of HFOs separately. We defined ripples as any eight consecutive peak points with absolute amplitudes >3.5 SD from the baseline mean amplitude, of which six peak points were more than nine SD above the baseline mean amplitude. FRs were identified as any eight consecutive peak points with absolute amplitudes >3 SD above the baseline mean amplitude, of which four peak points were more than 10.5 SD above the baseline mean amplitude. After HFO detection, 5 s original EEG epochs centered on the HFOs were automatically extracted. The epochs were categorized as EZ or non-EZ HFOs according to the resection scope. Before deep learning, the same number of ripples or FRs epochs were selected randomly from the EZ and non-EZ data from all patients. Of these, 70% were made the training dataset, 15% were used in the validation dataset, and 15% became the test dataset. The training and validation datasets were used to train and adjust the parameters, whereas the test dataset was used to test the network accuracy.

### Classification of High-Frequency Oscillations Using Convolutional Neural Networks

**Figure 1:**
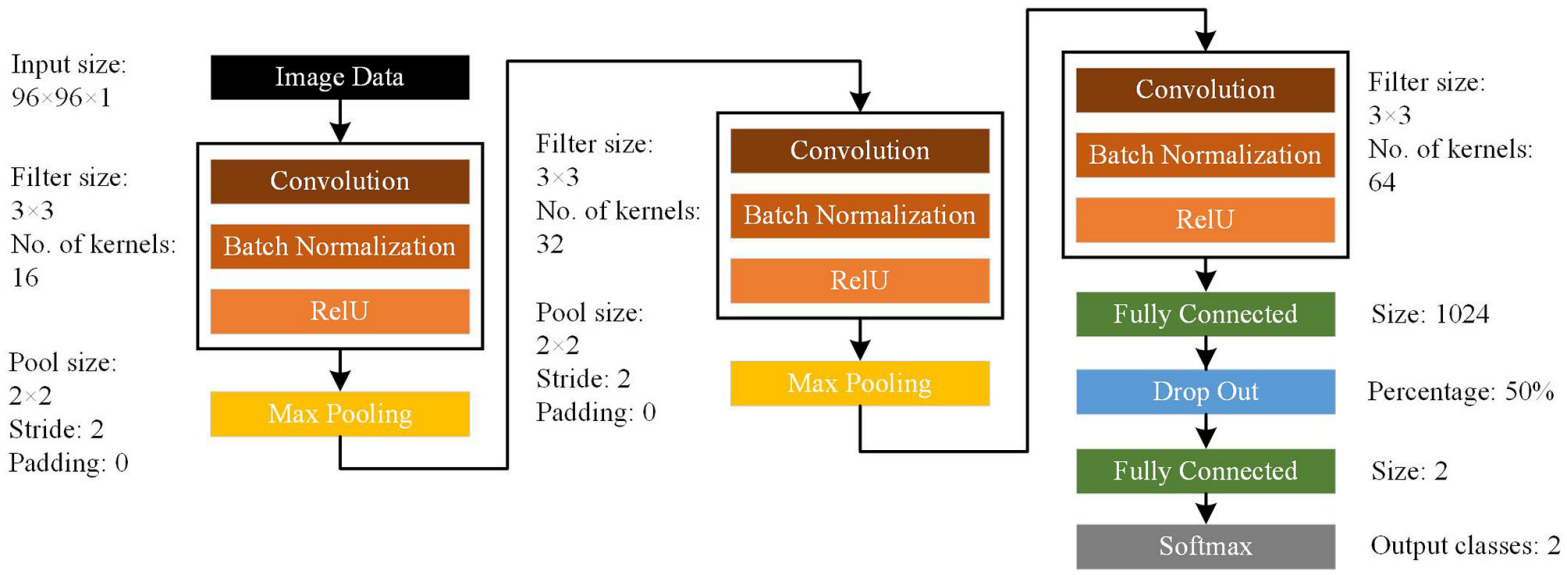
Architecture of the convolutional neural network (CNN) model. The CNN model consists of input, convolutional, batch normalization, max pooling, fully connected, dropout, and softmax layers.

CNN represents a common deep learning method with relatively few parameters and good performance (22, 23). Therefore, we chose this type of deep learning structure for data classification. The network structure is shown in Figure 1. Our CNN model consisted of input, convolutional, batch normalization, max pooling, fully-connected, dropout, and softmax layers. For ripples, the original images were 200 × 120 × 1 pixel. For FRs, the original images were 400 × 300 × 1 pixel. Then we used bicubic interpolation method to resize images to 96 × 96 × 1 pixel for use as input data (“imresize” function in Matlab 2019b). The training images used in this study included four types of transformations. The EZ and non-EZ HFOs were defined as “0” and “1,” respectively. Three sets of convolution and max pooling layers were adopted to improve the classification accuracy. Since the Rectified Linear Unit (ReLU) function has good non-linear mapping characteristics, it was adopted uniformly as the transformation function. The rate of initial learning during network training, was set to 0.01. The validation and test data sets were also converted into 96 × 96 × 1 pixel images and used as input to the trained network. The final test data outputs were again labeled “0” or “1” to distinguish the classification results. Then, the actual and output labels were compared. The identification was correct if the test data actual and output labels matched. Otherwise, it was wrong. Finally, this process was performed for 100 rounds to measure the stabilizing effect of the network and determine the classification accuracy.

Our null hypothesis was that the classification is random. This null hypothesis could be rejected when the accuracy of the CNN network to classify EZ and non-EZ HFOs is higher than 50%.

### Electroencephalogram Feature Transformation

Because the EEG data were one-dimensional, they could not be input directly to the CNN. Transformation of the EEG data into two-dimensional images was required. In our study, the classification accuracy of CNN using various two-dimensional images were compared. The EEG data were converted into four types of images: original waveforms, filtered waveforms, wavelet spectrum images, and smoothed pseudo Wigner–Ville distribution spectrum images.

#### Original Waveform

The original waveform is one of the most direct and commonly used two-dimensional image transformation methods. First, since the durations of most HFOs were <1 s, and FRs were usually shorter than ripples (24, 25), 200 ms (for ripples) or 100 ms (for FRs) epochs centered on each HFO were extracted from the original EEG data. This transformation used two scales to draw the EEG. In the fixed scale, the amplitude scale of the EEG signal was within ±1,000 μ*V* when an EEG epoch was drawn. This scale could restore the morphology of the original data well. In the fitted scale, the maximum absolute EEG signal value α μ*V* was calculated. Then, the EEG amplitude scale was within ±α μ*V* when the signal was drawn. This scale could effectively eliminate differences between EEG signal baseline conditions caused by different references or equipment.

#### Filtered Waveform

The filtered waveform is a two-dimensional image transformation method whose result is most similar to EEG morphology when manually analyzing HFOs. First, the data were filtered using a finite impulse response (FIR) filter with a hamming window. They were band-passed by 80–200 Hz for ripples with window length of 330 points and 200–500 Hz for FRs with window length of 132 points. Then, 200 ms (for ripples) or 100 ms (for FRs) HFO-centered filtered EEG epochs were extracted. Again, two types of scales were used to draw the filtered signal. In the fixed scale, the amplitude scale of the ripple was within ±50 μ*V* and the FR was within ±10 μ*V* when an HFO segment was plotted. The fitted scale process was the same as that used with the preceding original waveform.

#### Wavelet Spectrum Image

Time-frequency spectrum images can transform one-dimensional EEG signals from the time domain to the time-frequency domain and are widely used in EEG signal processing. The most popular algorithms include short-time Fourier transforms, wavelet transforms, and empirical-mode decomposition. In this study, a Morlet wavelet with a center frequency of eight was used for analysis (12). Ripples were drawn with a frequency range of 80–200 Hz and a step size of 1 Hz. FRs were drawn with a frequency range of 200–500 Hz and a step size of 1 Hz. In order to remove the influence of boundary effects on the wavelet graph, wavelet spectra of 1,000 ms epochs centered on the extracted 5 s data were calculated. Then, partial wavelet spectrum graphs with durations of 200 ms (for ripples) or 100 ms (for FRs) centered on the 5 s graphs were restored.

The scales were similar to those of other transformations. In the fixed scale, the wavelet spectrum energy mapping range was within 0–1,000 μ*V*^2^ for ripples and within 0–10 μ*V*^2^ for FRs when the wavelet spectrum image was plotted. In the fitted scale, the maximum absolute value α μ*V*^2^ of wavelet spectrum was computed. When the wavelet spectrum graph was drawn, the wavelet spectrum energy scale was within 0 α μ*V*^2^.

#### Smoothed Pseudo Wigner–Ville Distribution Spectrum Image

The Wigner–Ville distribution is widely used in the field of time-frequency feature extraction. It is a powerful, appealing tool for the analysis of non-stationary, non-linear, and transient signals (26). This method has higher temporal and frequency resolutions than wavelet transforms. However, cross-term interference occurs when this algorithm is applied to signals with multiple frequency components. We used a smoothed pseudo Wigner–Ville distribution (SPWVD) to reduce this influence. The SPWVD improves on WVD by using windowed smoothing for time-frequency analysis. This method can reduce interference from cross terms with little loss in time and frequency resolution, and is thus more suitable for EEG analysis.

The central point of each HFO was set as time 0, and data from 500 ms before and after this point were used for SPWVD analysis. This study used a Kaiser window with a length of 1,000 ms and a frequency of 1,000 Hz to smooth in time and frequency. For a continuous signal *x*(*t*), the WVD is defined as:


(1) $$\begin{array}{r} WVD_{x\;}\left(t,f\right)=\int_{-\infty }^{\infty }x\left(t+\frac{\tau }{2}\right)x^{*}\left(t-\frac{\tau }{2}\right)e^{-j2\pi f\tau }\;d\tau . \end{array}$$

For a discrete signal with *N* samples, the distribution becomes:


(2) $$\begin{array}{r} WVD_{x\;}\left(n,k\right)=\overset{N}{\underset{m=-N}{\sum }}x\left(n+m/2\right)x^{*}\left(n-m/2\right)e^{-j2\pi km/N}. \end{array}$$

The SPWVD used independent windows to smooth in time and frequency:


(3) $$\begin{array}{r} SPWVD_{x}^{g,H\;}\left(t,f\right)=\int_{-\infty }^{\infty }g\left(t\right)H\left(f\right)x\left(t+\frac{\tau }{2}\right)x^{*}\left(t-\frac{\tau }{2}\right)e^{-j2\pi f\tau }\;d\tau . \end{array}$$

where *g*(*t*) is the smoothed window of time domain, and *H*(*f*) is the smoothed window of frequency domain.

Since the frequency components of the EEG signals follow power laws, the energy in the time-frequency SPWVD diagram decays rapidly as the frequency increases. Therefore, it is difficult to distinguish high-frequency information in the SPWVD time-frequency spectrum. We used the following improvements to solve this problem.

**Figure 2:**
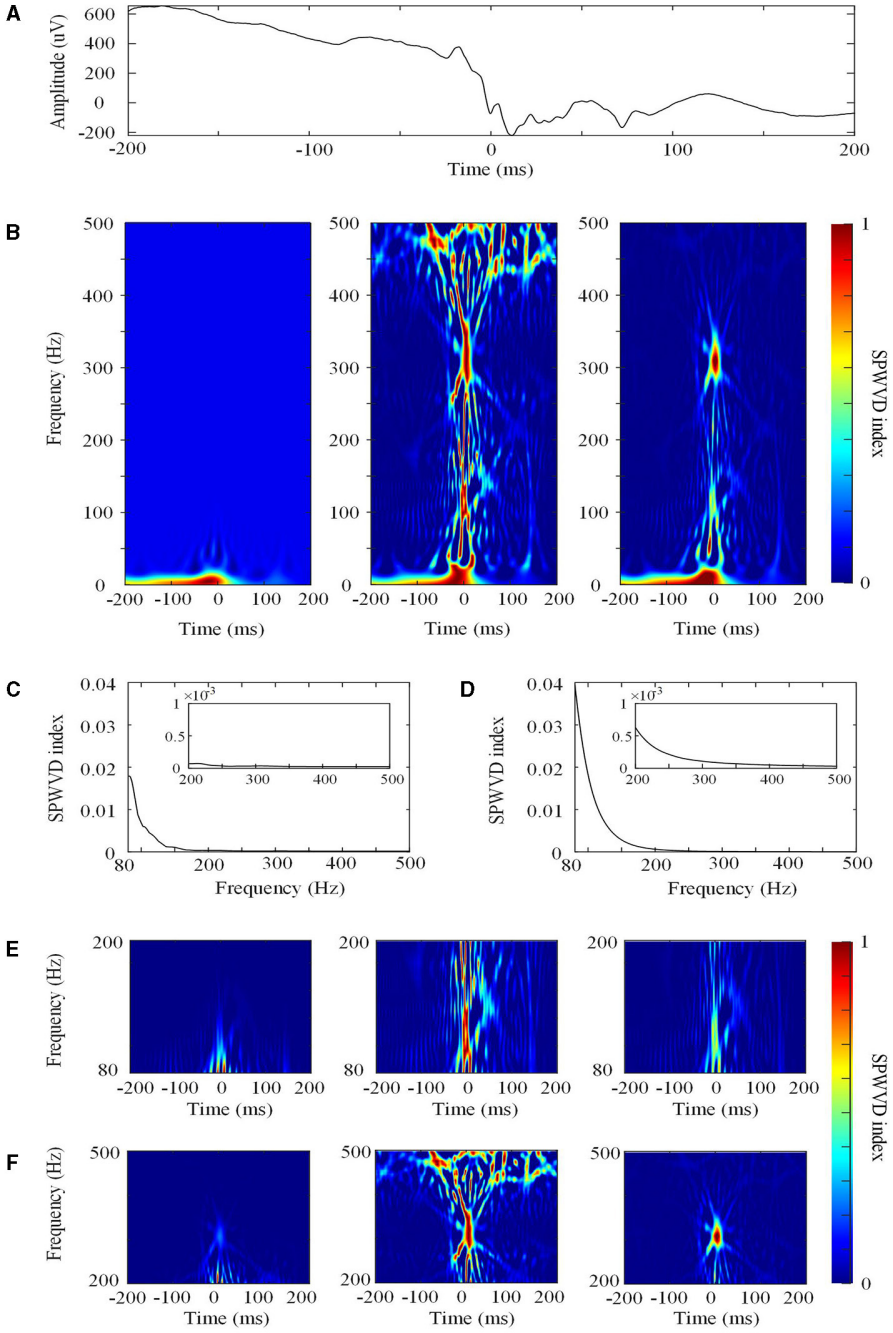
The smoothed pseudo Wigner–Ville distribution (SPWVD). **(A)** The original waveform of a 400 ms epoch. **(B)** Left: the first normalization of the SPWVD transformation (0–500 Hz). In this figure, all of the SPWVD transformation values are normalized by dividing all time points by the maximum SPWVD value. The normalized value (Ŵ_*tf*_) is the SPWVD index. Middle: Re-normalized SPWVD. The Ŵ_*tf*_ in the left is re-normalized by dividing all time points in each HFO by the maximum SPWVD index in all frequencies. Right: Adjusted SPWVD (ASPWVD). The Ŵ_*tf*_ in the left were adjusted by dividing all HFOs time points by the median maximum SPWVD index. **(C)** Curve C for re-normalized SPWVD. This is curve C for this epoch from 80 to 500 Hz. The small rectangle in the figure is the enlargement of curve C for FR (200–500 Hz). **(D)** Curve C for the ASPWVD. This is curve C with the median maximum SPWVD index for all epochs from 80 to 500 Hz. The small rectangle in the figure is the enlargement of curve C for FR (200–500 Hz). **(E)** Ripple (80–200 Hz) SPWVD spectrum images. Left: the first normalization of the SPWVD spectrum image. Middle: Re-normalized SPWVD spectrum image. Right: the ASPWVD spectrum image. **(F)** FR (200–500 Hz) SPWVD spectrum image. Left: the first normalization of SPWVD spectrum image. Middle: Re-normalized SPWVD spectrum image. Right: the ASPWVD spectrum image. **(E,F)** indicate that the ASPWVD spectrum image provides higher time frequency resolution and less noise than the re-normalized SPWVD.

First, for the specified frequency *f*, the SPWVD transformation was normalized by dividing by the maximum SPWVD value at all timepoints (Figures 2A,B). The normalized SPWVD transformation value was named the SPWVD index. The formulas are as follows:

(4) $$\begin{array}{r} {Ŵ}_{tf}=\{\begin{array}{c} \;0,\;W_{tf}\leq 0\\ \frac{W_{tf}}{\max\left(M_{f}\right)},W_{tf}>0 \end{array} \end{array}$$

where Ŵ_*tf*_ is the SPWVD transformation of HFO; *t* is time, which ranges from 0 to 1,000 ms; *f* is frequency, which ranges from 0 to 1,000 Hz; and *M*_*f*_ is the curve that contains the SPWVD transformation values and time information for a specific frequency *f*. The sample frequency is 2,000 Hz and the frequency interval is 0.5 Hz.

Second, we innovatively applied two further improvements to the algorithms in this study:

(1) The re-normalized SPWVD. After computing the normalized *W*_*tf*_ values for all of the frequencies (80–200 or 200–500 Hz) in an HFO, we obtained a curve C for each HFO and determined the maximum SPWVD index in the curve (Figure 2C). Then, the *W*_*tf*_ was re-normalized by dividing by the maximum SPWVD index at all frequencies for each HFO.

(2) The adjusted SPWVD (ASPWVD). Although SPWVD provides an improvement by adding windowed smoothing to the WVD, noise remains in the EEG. This can decrease the accuracy of time-frequency analysis. Therefore, we determined the median of the maximum SPWVD indexes of the C curves of SPWVD transformation for all HFOs (Figure 2D). Then, the SPWVD transformations of all of the HFOs were adjusted by dividing them by the median maximum SPWVD index. After adjustment, the WVD-related noise was suppressed and the features of the time-frequency were highlighted (Figures 2E,F). ASPWVD transformed images could obtain oscillation energy better while retaining their time frequency resolution.

Then, partial SPWVD spectrum graphs with durations of 200 ms (for ripples) or 100 ms (for FRs) that were centered on the 1,000 ms graphs were restored.

### Statistical Analysis

In the current study, we sought to detect epileptic HFOs in EZs from HFOs in non-EZs. Therefore, true positive (TP) refer to cases where HFOs in EZs were classified correctly and false negative (FN) refer to cases where HFOs in EZs were classified as HFOs in non-EZs. False positive (FP) refers to cases where HFOs in non-EZs were classified as HFOs in EZs and true negative (TN) refer to cases where HFOs in non-EZs were classified correctly. The sensitivity, specificity, and accuracy are defined in the following equations:

(5) $$\begin{array}{r} \begin{array}{c} \text{Sensitivity}=\frac{\text{TP}}{\text{TP}+\text{FN}}\;\times 100\\ \text{Specificity}=\frac{\text{TN}}{\text{TN}+\text{FP}}\;\times 100\\ \text{Accuracy}=\frac{\text{TP}+\text{TN}}{\text{TP}+\text{FN}+\text{TN}+\text{FP}}\;\times 100 \end{array} \end{array}$$

All data are expressed as mean ± SD and were analyzed using Sigmaplot version 12.0 (Systat Software, Inc, San Jose, CA, USA). The one-way ANOVA was used to compare the accuracies of CNN networks using four types of transformation images. The Tukey test was used for pairwise comparisons. The *t*-test and Mann–Whitney rank sum test were used for normal distribution data and non-normal distribution data to compare the CNN classification results based on fixed-scale and fitted-scale images. A *p* < 0.05 was considered statistically significant.

## Results

A total of 19 subjects with focal refractory epilepsy were included in the study (10 females). Their demographics and clinical characteristics are shown in Table 1. A total of 20,238 ripples (12,756 in EZs and 7,482 in non-EZs) and 5,189 FRs (3,146 in EZs and 2,043 in non-EZs) were detected automatically. Therefore, before deep learning, 7,000 ripple epochs and 2,000 FR epochs were selected randomly from the EZ and non-EZ groups for analysis.

**Table 1:** Demographics and clinical characteristics.

**ID**	**Gender**	**Age, years**	**MRI**	**Implantation sites**	**Removed sites**
1	Male	16	L HS	L T, L LF, L H, L A	L AT, L partial H, L A
2	Male	33	Abnormal signal in R F	R MFG, R PreG, R PosG, R SFG, R Pos T, R P O	R Pos T, R P O
3	Female	25	Old bleeding foci in L P O	L F T, L T P	L P, L Wernicke zone
4	Female	14	Normal	L SFG, L MFG, L IFG, L M F, L T, L H	L SFG, L MFG, L IFG, L M F
5	Male	12	Abnormal signal in L F, L P cortex	L Lat F, L B F, L T, L I	L Lat F, L B F
6	Female	24	Normal	L M F, L SFG, L PreG, L P, L B F, L Lat F, L F, L M F	L M F, L SFG, L Pos T, L P
7	Male	11	Bilateral HS	R P, R O, R P O,	R P, R O
8	Male	35	Normal	R F, R SFG, R B F, R T, RH, R A	R F, R SFG, R B F, R AT, R H, R A
9	Female	42	R HS, bilateral CA	R T, R F P, bilateral H, bilateral A	R AT, R H, R A
10	Male	24	Normal	L F, L T, L A, L H	L F, L AT
11	Female	39	Normal	Bilateral F, bilateral T, bilateral H, bilateral A	L AT, L partial H, L Lat OG
12	Male	21	Normal	Bilateral SFG, bilateral MFG, bilateral IFG, bilateral M F, bilateral B F, bilateral T, bilateral B T	L F, L M F
13	Female	36	OPCA	R L F, R M F, R B F, R T, R P, R H, R A	R Lat F, R M F, R B F
14	Male	16	Atrophy in L H and the whole cortex	Bilateral F, R B F, bilateral T, R I, L OFC, R H, R A	R B F, R I
15	Female	26	Bilateral CA	L F P, L middle T, L Pos T, L P O	L Pos T, L P O
16	Male	20	Encephalomalacia foci in L T P O	L F, L T, L P, L O	L P O
17	Female	8	Normal	R F, R O, R P, R T, R M F, R H	R P, R O
18	Female	27	Normal	L F, L P, L T, L H, L A	L F P, L Pos T
19	Female	13	AC in L T and L LF, abnormal signal in bilateral OHLV	R CS, R P, R P O, R T	R O

*L, left; HS, hippocampal sclerosis; T, temporal; LF, lateral fissure; H, hippocampus; A, amygdala; AT, anterior temporal; R, right; F, frontal; MFG, middle frontal gyrus; PreG, precentral gyrus; PosG, postcentral gyrus; SFG, superior frontal gyrus; Pos, posterior; P, parietal; O, occipital; IFG, inferior frontal gyrus; M, mesial; Lat, lateral; B, basis; CA, cerebellar atrophy; OG, orbitofrontal gyrus; OPCA, olivo-ponto-cerebellar atrophy; I, insular; OFC, orbit frontal cortex; AC, arachnoid cyst; OHLV, occipital horn of lateral ventricle; CS, central sulcus*.

**Figure 3:**
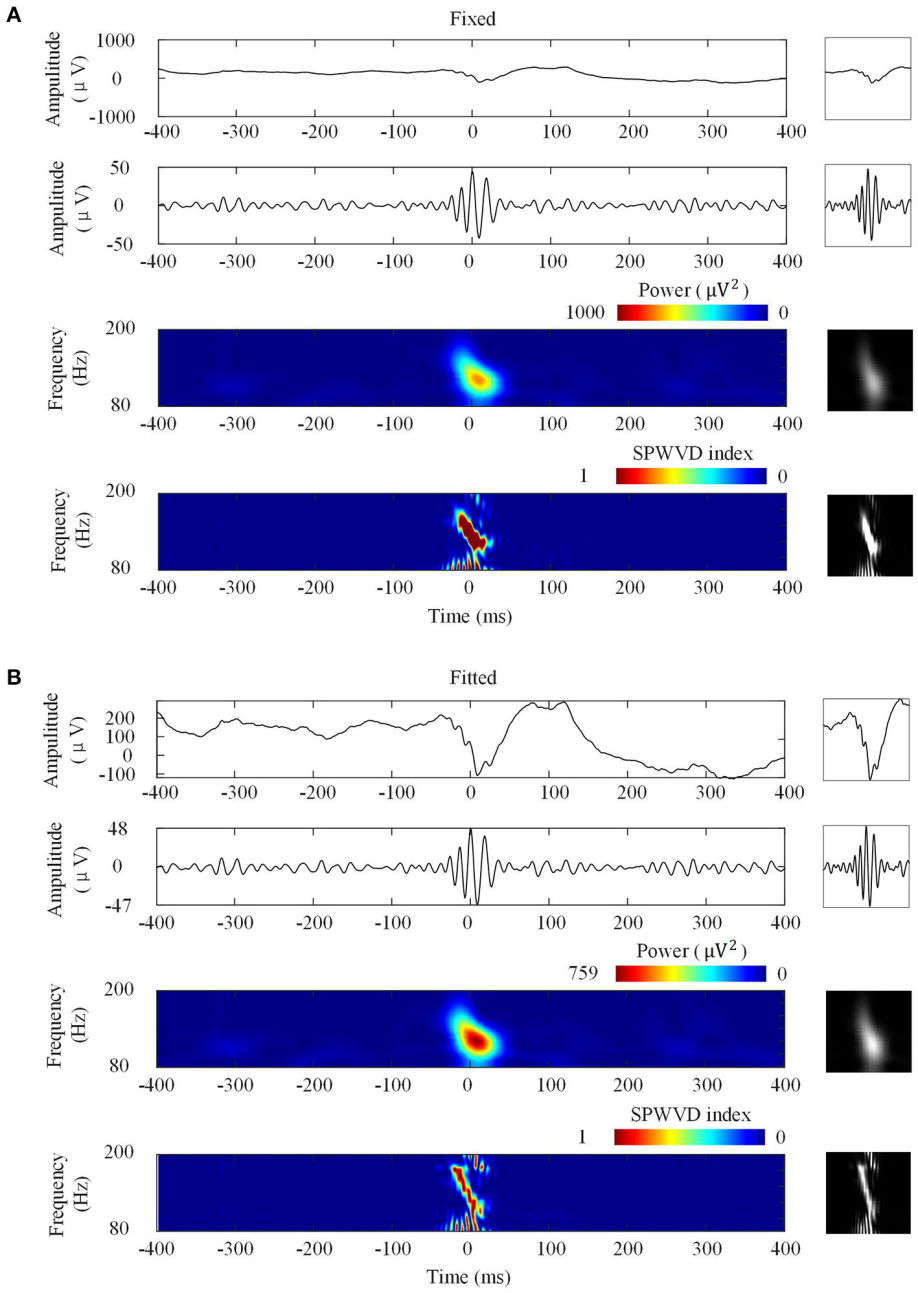
Four ripple image transformations. Left: Transformations of a ripple. Right: Network inputs in the form of two-dimensional 96 × 96-pixel images that represent various transformations. **(A)** Transformations with fixed scales. From top to bottom are original waveforms, filtered (80–200 Hz) waveforms, wavelet spectrum images, and ASPWVD spectrum images. **(B)** Transformations with fitted scales. From top to bottom are original waveforms, filtered (80–200 Hz) waveforms, wavelet spectrum images, and re-normalized SPWVD images.

**Figure 4:**
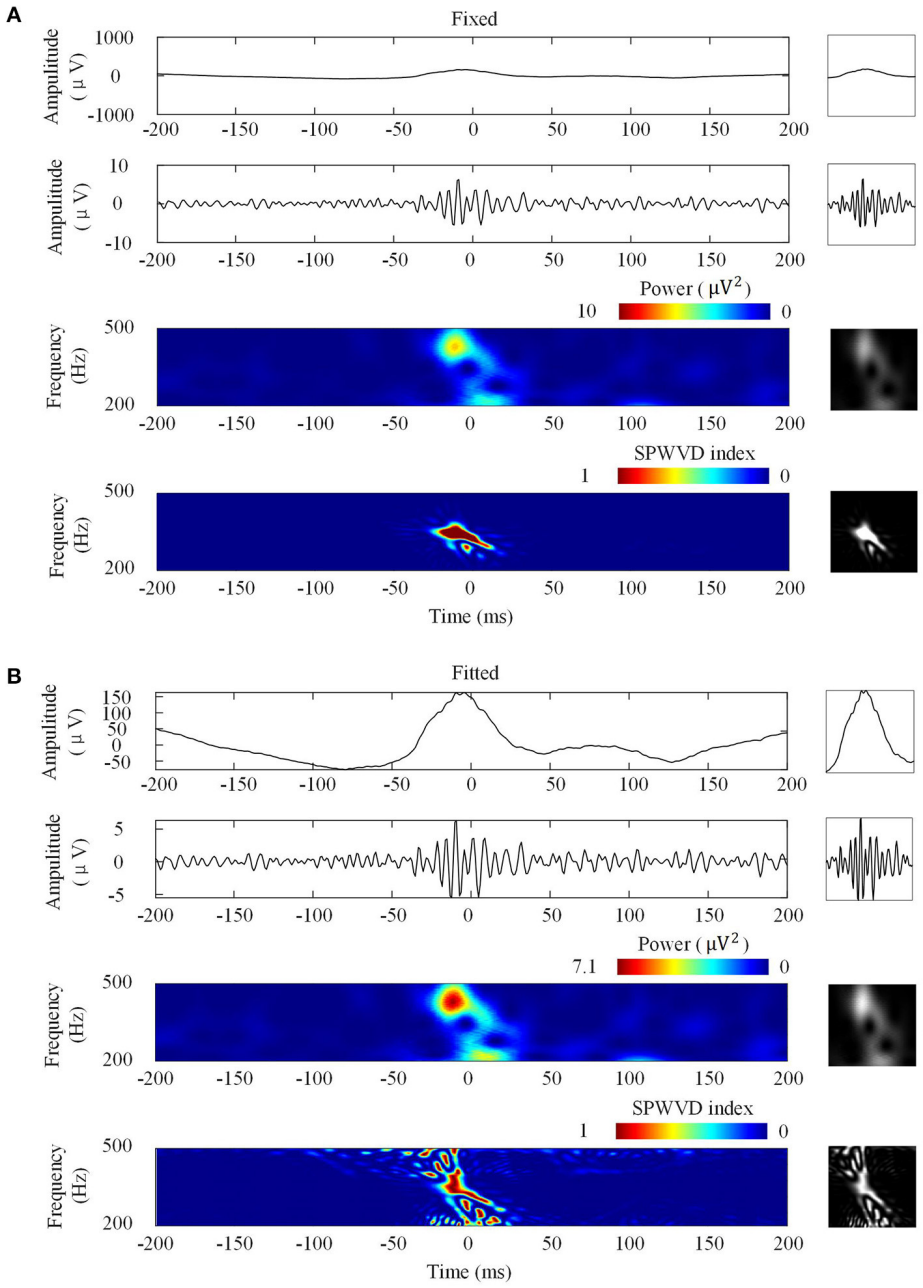
Four fast ripples (FRs) image transformations. Left: Transformations of a FR. Right: Network inputs in the form of two-dimensional pictures of 96 × 96 pixels that represent various transformations. **(A)** Transformations with fixed scales. From top to bottom are original waveforms, filtered (200–500 Hz) waveforms, wavelet spectrum images, and ASPWVD spectrum images. **(B)** Transformations with fitted scales. From top to bottom are original waveforms, filtered (200–500 Hz) waveforms, wavelet spectrum images, and re-normalized SPWVD spectrum images.

**Figure 5:**
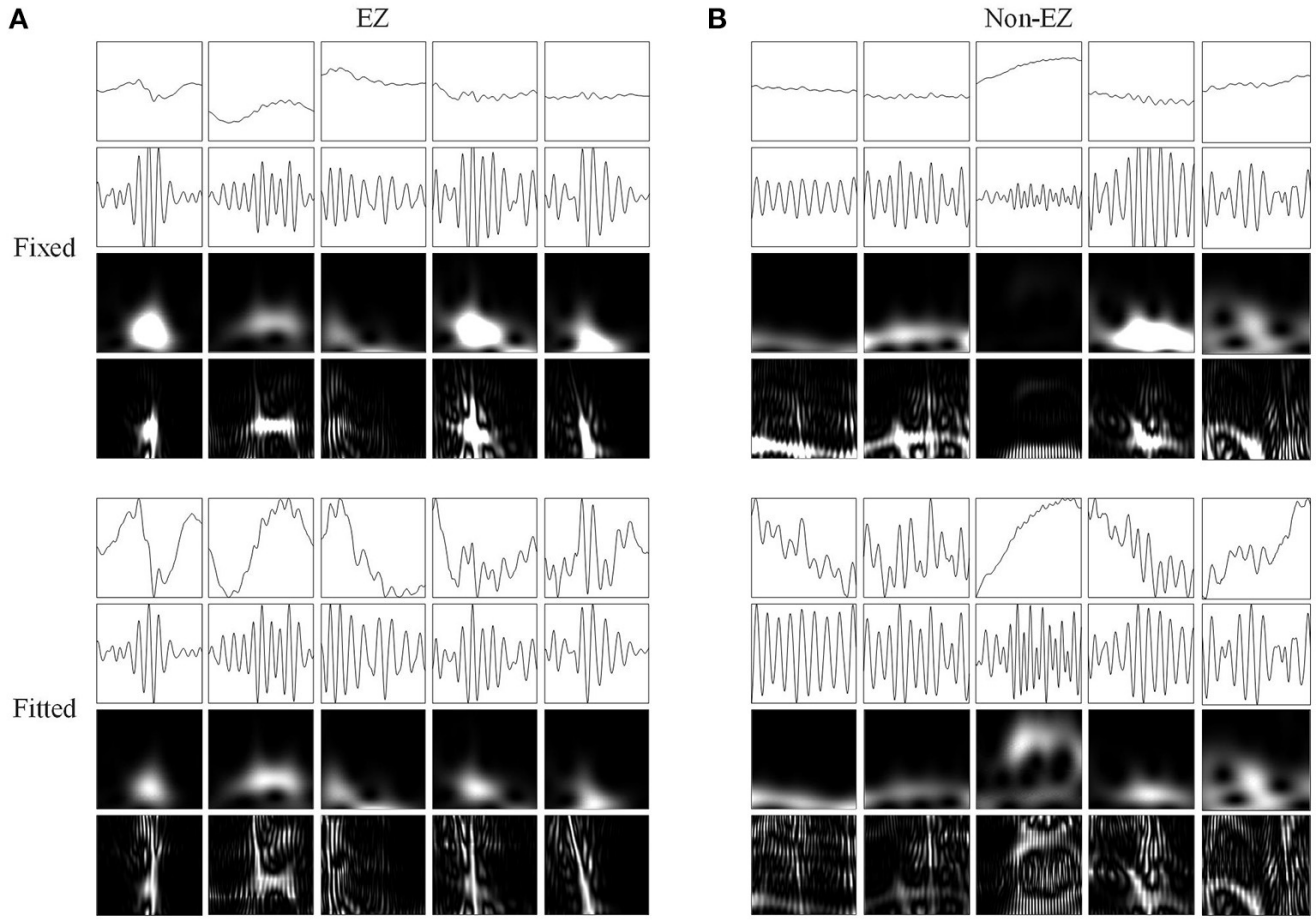
Network inputs in the form of two-dimensional pictures of ripples. **(A)** Two-dimensional pictures (96 × 96 × 1 pixel) of five ripples in different channels of EZs. Upper and lower four panels are fixed and fitted scales. The four panels are original waveforms, filtered (80–200 Hz) waveforms, wavelet spectrum images, and SPWVD spectrum images. **(B)** Two-dimensional pictures (96 × 96 × 1 pixel) of ripples in different channels of non-EZs. Upper and lower four panels are fixed and fitted scales. The four panels are original waveforms, filtered (80–200 Hz) waveforms, wavelet spectrum images, and SPWVD spectrum images.

**Figure 6:**
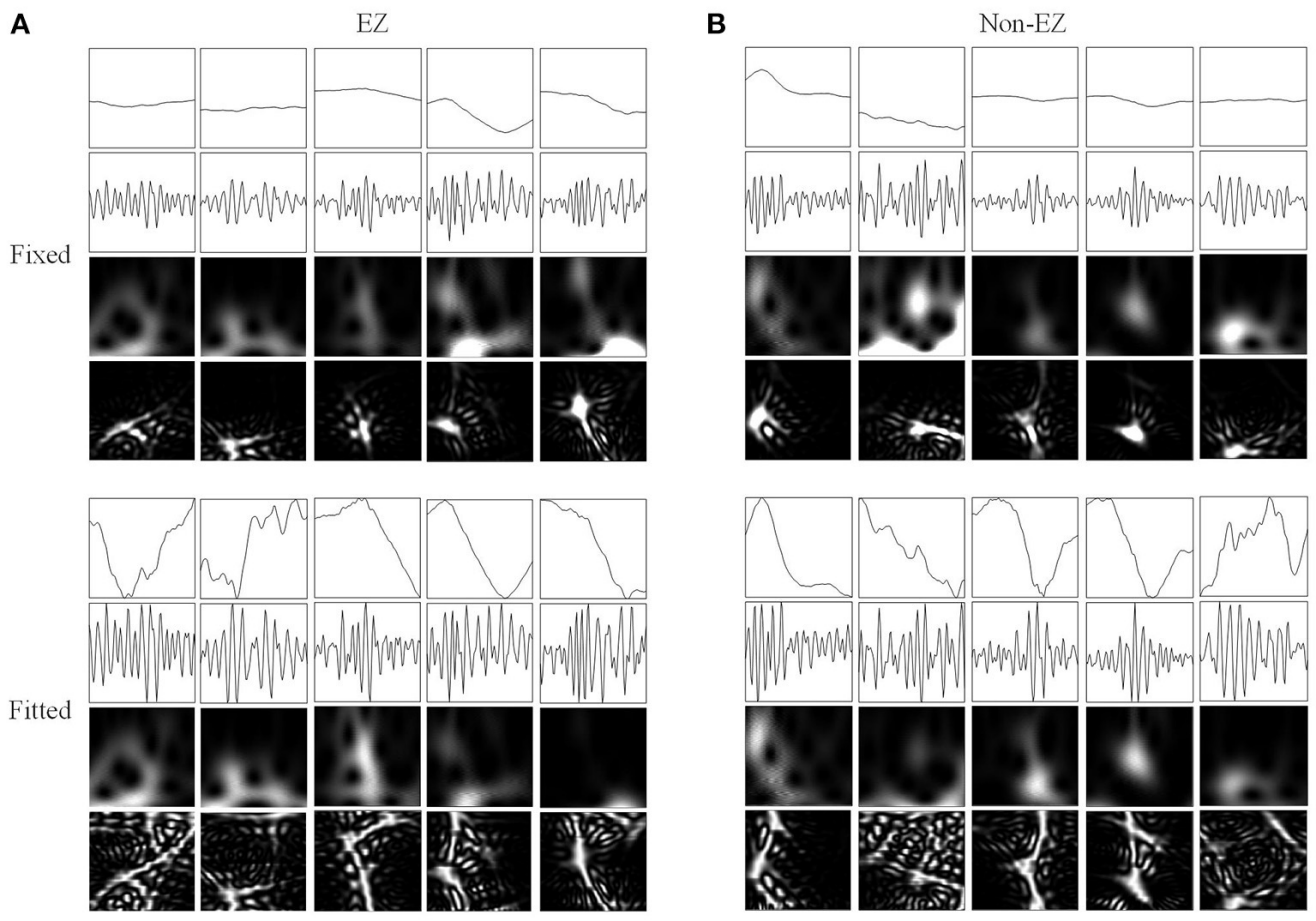
Network inputs in the form of two-dimensional pictures of FRs. **(A)** Two-dimensional pictures (96 × 96 × 1 pixel) of five FRs in different channels of EZs. Upper and lower four panels are fixed and fitted scales. The four panels are original waveforms, filtered (200–500 Hz) waveforms, wavelet spectrum images, and SPWVD spectrum images. **(B)** Two-dimensional pictures (96 × 96 × 1 pixel) of ripples in different channels of non-EZs. Upper and lower four panels are fixed and fitted scales. The four panels are original waveforms, filtered (200–500 Hz) waveforms, wavelet spectrum images, and SPWVD spectrum images.

Figure 3 shows the four types of ripple transformations with two scales. The re-normalized SPWVD image displays higher time-frequency resolution than the wavelet spectrum image. The ASPWVD further suppresses the WVD-related noise and highlights the features of HFOs. Figure 4 shows four types of FR transformations with two scales and excellent two-dimensional ASPWVD images. Figures 5, 6 show two-dimensional 96 × 96 × 1 pixel images of five different ripples or FRs from EZ and non-EZ that were put into the network.

**Figure 7:**
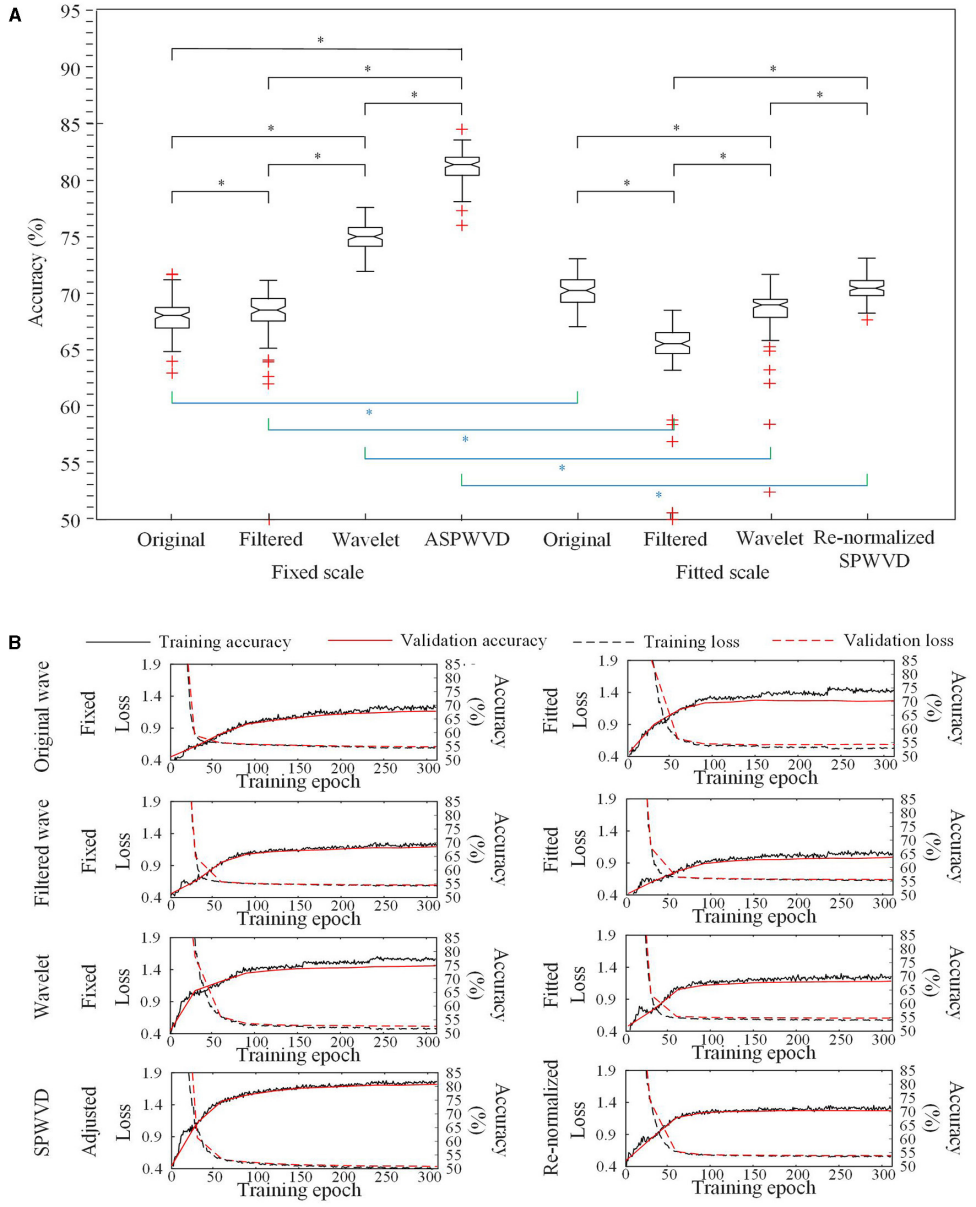
Convolutional neural networks (CNN) accuracies for ripples classification. **(A)** Accuracies of CNNs for ripples classification using various transformations. The CNN network using the fixed-scale ASPWVD images provides the highest accuracy (*p* < 0.05). Except for original ripple waveforms, the results from CNN models that are trained using fixed-scale images are significantly better than those from models trained using fitted-scale images (*p* < 0.05). **(B)** CNN training and validation accuracies and losses are displayed. Asterisk (*) means a statistically significant difference.

**Figure 8:**
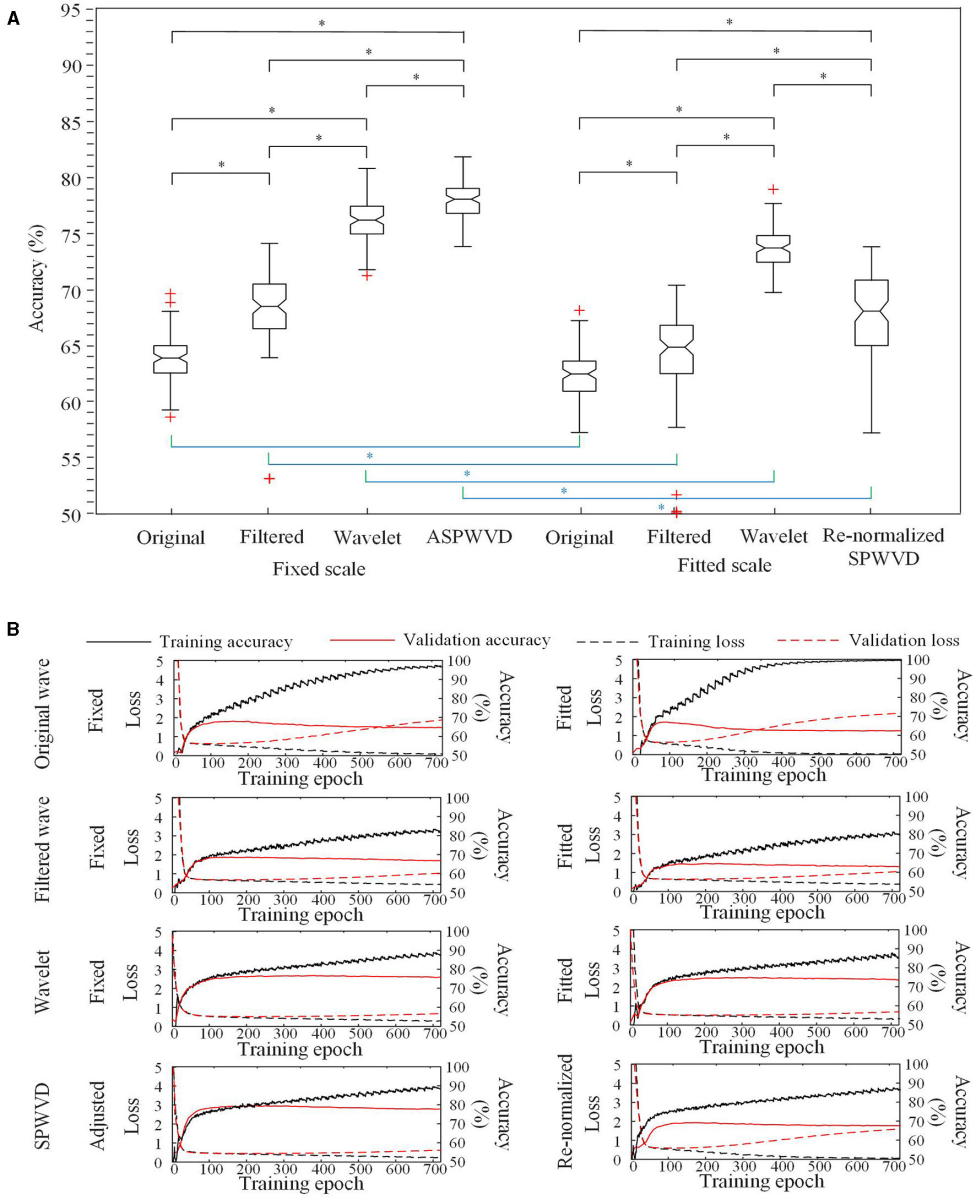
CNN accuracies for FRs classification. **(A)** Accuracies of CNNs for FRs classification using various transformations. The CNN using the fixed-scale ASPWVD images provides the highest accuracy (*p* < 0.05). The results from CNN models that are trained using fixed-scale images are significantly better than those from models trained using fitted-scale images (*p* < 0.05). **(B)** CNN training and validation accuracies and losses are displayed. Asterisk (*) means a statistically significant difference.

The classification performances of CNN models trained on four different transformations of EEG features with two scales are shown in Table 2. The accuracies of the CNN networks to classify EZ and non-EZ HFOs were higher than 50%. Therefore, the null hypothesis that the classification is random was rejected. Except for original ripple waveforms, the results from the CNN models that are trained using fixed-scale images are significantly better than those from models trained using fitted-scale images (*p* < 0.05, Figures 7, 8). Of the four models based on fixed-scale transformations, the CNN model based on the ASPWVD exhibits the best accuracies (80.89 ± 1.43% and 77.85 ± 1.61% for ripples and FRs, respectively, *p* < 0.05). The training and validation accuracies and losses of ripples and FRs are also displayed in Figures 7, 8.

**Table 2:** Performance of convolutional neural networks (CNNs) trained using various electroencephalogram (EEG) data transformations.

**HFO**	**Transformation method**	**Fixed scale /ASPWVD^a^** **(mean ± standard deviation)**			**Fitted scale/re-normalized SPWVD^b^** **(mean ± standard deviation)**		
		**Accuracy (%)**	**Sensitivity (%)**	**Specificity (%)**	**Accuracy (%)**	**Sensitivity (%)**	**Specificity (%)**
Ripple	Original waveform	67.90 ± 1.48	65.73 ± 1.52	73.45 ± 1.84	70.25 ± 1.27	69.93 ± 2.04	71.91 ± 1.47
	Filtered waveform	68.47 ± 4.13	68.36 ± 4.35	70.32 ± 4.49	63.99 ± 4.97	62.88 ± 5.52	65.76 ± 4.75
	Wavelet spectrum image	74.96 ± 1.19	72.37 ± 1.95	79.07 ± 2.05	68.07 ± 2.43	67.62 ± 2.32	69.56 ± 2.75
	SPWVD spectrum image	80.89 ± 1.43	80.10 ± 1.97	82.48 ± 2.25	70.48 ± 1.05	68.92 ± 2.59	73.53 ± 3.02
Fast ripple	Original waveform	64.09 ± 2.02	63.87 ± 2.24	64.49 ± 2.29	62.38 ± 1.95	62.46 ± 2.02	62.37 ± 2.16
	Filtered waveform	66.85 ± 6.04	65.81 ± 5.03	68.43 ± 4.98	62.84 ± 6.25	62.11 ± 5.36	63.20 ± 5.47
	Wavelet spectrum image	75.90 ± 1.81	76.40 ± 3.10	75.82 ± 3.33	73.60 ± 1.78	73.48 ± 2.85	73.75 ± 2.96
	SPWVD spectrum image	77.85 ± 1.61	77.39 ± 2.53	78.75 ± 2.33	67.80 ± 3.76	66.81 ± 4.10	68.32 ± 4.06

a *Adjusted smoothed pseudo Wigner–Ville distribution (ASPWVD)*;

b *Smoothed pseudo Wigner–Ville distribution (SPWVD)*.

## Discussion

In this study, a multi-feature CNN network structure was used to explore a new method of identifying EZ and non-EZ HFOs. ASPWVD images were applied creatively to transform one-dimensional EEG data into two-dimensional signals and help to improve the accuracy of the CNN network. As a result, the CNN model based on ASPWVD images provided the best accuracies with fixed scales (80.89 ± 1.43% and 77.85 ± 1.61% for ripples and FRs, respectively).

CNNs are used widely for EEG signal analysis and perform well (27–29). A traditional CNN structure was used to distinguish EZ and non-EZ HFOs in this study (30). We innovatively converted all epochs into four types of images with two scales. The CNN classification results produced by using networks that were trained using various EEG feature transformations indicated that fixed-scale image performance was usually significantly better than fitted-scale image performance. The fitted-scale images provided similar oscillation patterns of EZ and non-EZ HFOs. This led to ordinary classification performance. The better performance of CNNs that were trained using fixed-scale images indicates that there are potentially significant differences between the amplitudes of EZ and non-EZ HFOs. This finding is similar to previous results (31, 32).

The classification results from CNNs that were trained using ASPWVD transformed images were optimal. We applied this time-frequency analysis to transform one-dimensional EEG data into two-dimensional images. This succeeded because this method could reduce WVD-related noise and receive higher time-frequency analysis resolution than wavelet transformation. Therefore, more detailed time and frequency information of HFOs in the image was analyzed during convolution with the CNN than other transformations. This could help the CNN to identify oscillations more accurately. It is widely proven that physiological and pathological HFOs exhibit overlapping properties (33, 34), and different brain regions generate highly variable rates of physiological HFOs (7, 35). The differences between EZ and non-EZ HFOs are reflected mainly in the oscillation details. Thus, a method that adequately expresses oscillation details, such as the ASPWVD, can effectively assist CNN model in classification of EZ and non-EZ HFOs.

Our results indicate that CNNs can distinguish EZ and non-EZ HFOs, but the highest accuracy achieved is limited to 81%. Apart from the limitations of the method, this limited accuracy may be related to the inherent characteristics of the data for two reasons. First, the pervasiveness of physiological HFOs limits accuracy. Due to the presence of functional neural networks in the brain, physiological HFOs are distributed widely among EZ and non-EZ areas. Therefore, certain characteristics may overlap EZ and non-EZ HFOs. This increases the difficulty of classifying HFOs in these two areas. Second, the blurred boundaries of real EZs may affect accuracy. Although the surgical outcomes of the patients in our group were Engel I and we defined the EZ according to the resection scope, the brain area that was resected during surgery might be larger than the real EZ range.

In addition, the number of patients that could be included was limited because of the restrictive inclusion criteria, and the training, validation, and test datasets were from the same patients because of the limited number of patients. This might cause higher accuracies than it would have been achieved in a scenario where the model was tested on patients that were different from the ones used in training and validation. Due to the small number of FRs, the performance of CNNs to classify EZ and non-EZ FRs were not as good as ripples. In the future, we will include more patients to further train and test the CNN model. Accurate EZ and non-EZ HFOs training datasets can help to train CNNs better. Furthermore, we hope to optimize the network structure and improve HFO characteristic transformation in order to modify our algorithm further.

## Conclusion

In this study, EZ and non-EZ HFOs were distinguished successfully using our multi-feature CNN model. Their classification was most effective when they used time-frequency information from EEG data that was filtered using the ASPWVD method. We innovatively proposed an optimized CNN model to classify EZ and non-EZ HFOs, though the accuracy of our CNN model is not yet sufficient for clinical use. In the future, accurate training datasets that distinguish physiological and pathological HFOs or occurrence of other network structures may enable deep learning networks to produce a simple, fast, and reliable system for classifying EZ and non-EZ HFOs.

## Data Availability Statement

The raw data supporting the conclusions of this article will be made available by the authors, without undue reservation.

## Ethics Statement

The studies involving human participants were reviewed and approved by Research Ethics Board of Beijing Haidian Hospital. Written informed consent to participate in this study was provided by the participants' legal guardian/next of kin.

## Author Contributions

GR and JY contributed to designing the study, collecting, analyzing the data, and wrote a draft of the manuscript. All authors collected the data and interpreted the results and reviewed and revised the article and confirmed its final version.

## Funding

The study was financially supported by the National Natural Science Foundation of China (81801280 to GR, 61803003 to JY, 81601126 to JR, 81971202 to XY), the Youth Research Fund of Beijing Tiantan Hospital affiliated to Capital Medical University (2017-YQN-01 to GR), the National Key R&D Program of China grant (2017YFC1307500 to QW), the Capital Health Research and Development of Special grants (2016-1-2011 and 2020-1-2013 to QW), the Beijing-Tianjin-Hebei Cooperative Basic Research Program (H2018206435 to QW), and the Beijing Natural Science Foundation (Z200024 to QW).

## Conflict of Interest

The authors declare that the research was conducted in the absence of any commercial or financial relationships that could be construed as a potential conflict of interest.

## Publisher's Note

All claims expressed in this article are solely those of the authors and do not necessarily represent those of their affiliated organizations, or those of the publisher, the editors and the reviewers. Any product that may be evaluated in this article, or claim that may be made by its manufacturer, is not guaranteed or endorsed by the publisher.
